# CD39-Expressing CD8^+^ T Cells as a New Molecular Marker for Diagnosis and Prognosis of Esophageal Squamous Cell Carcinoma

**DOI:** 10.3390/cancers15041184

**Published:** 2023-02-13

**Authors:** Meitong Liu, Yaning Zhao, Zhuoyun Xiao, Rongmiao Zhou, Xiaodong Chen, Saijin Cui, Shiru Cao, Xi Huang, Tianyu Chen, Xiangran Huo, Guoqiang Zhang, Ziqiang Tian, Na Wang

**Affiliations:** 1 Cancer Institute, The Fourth Hospital of Hebei Medical University, Shijiazhuang 050011, China; 2 College of Public Health, Hebei Medical University, Shijiazhuang 050031, China; 3 Department of Dermatology, The First Hospital of Hebei Medical University, Shijiazhuang 050000, China; 4 Candidate Branch of National Clinical Research Center for Skin Diseases, Shijiazhuang 050000, China; 5 Department of Thoracic Surgery, The Fourth Hospital of Hebei Medical University, Shijiazhuang 050011, China

**Keywords:** esophageal squamous cell carcinoma, CD39^+^CD8^+^ T cells, diagnosis, prognosis, nomogram, tumor microenvironment

## Abstract

**Simple Summary:**

Esophageal squamous cell carcinoma (ESCC) is one of the most common cancers in the world with an insidious onset and poor prognosis. Studies have reported that CD39 is highly expressed on several human tumors and is closely associated with CD8^+^ T cells in the tumor microenvironment. We aimed to explore the effect of CD39 expression on CD8^+^ T cells and on the diagnosis and prognosis of ESCC by analyzing 95 ESCC samples in order to better predict the probability of patients’ survival by establishing a nomogram model. We observed that CD39 expression was higher on CD8^+^ T cells in cancer tissues. High CD39-expressing CD8^+^ T cells were an independent risk factor for the prognosis of ESCC, and its expression was significantly positively correlated with the expression of *PDCD1*, *CTLA4*, and *HAVCR2*. It is further proposed that the restoration of partially exhausted CD8^+^ T cells by inhibiting CD39 may be a new strategy for treating ESCC.

**Abstract:**

We aimed to explore the effect of CD39 expression on CD8^+^ T cells and on the diagnosis and prognosis of esophageal squamous cell carcinoma (ESCC). The independent prognostic factors for the surgical specimens of the 95 ESCC patients were screened by multivariate Cox regression analysis. Differential gene expression analysis was performed by the NetworkAnalyst platform based on data from the Gene Expression Omnibus (GEO). The expression of CD39 on CD8^+^ T cells in the CK^+^ region was higher in cancer tissue than in paracancerous tissue (*p* = 0.011), and high CD39-expressing CD8^+^ T cells in the CK^+^ region (HR, 2.587; *p* = 0.033) and high CD39-expressing CD8^+^ T cells in the CK^−^ region (HR, 3.090; *p* = 0.008) were independent risk factors for prognosis in ESCC patients; the expression of *ENTPD1* was upregulated in ESCC tissues compared to normal tissues (adjusted *p* < 0.001; log_2_ fold change = 1.99), and its expression was significantly positively correlated with the expression of *PDCD1*, *CTLA4*, and *HAVCR2*. High CD39-expressing CD8^+^ T cells can be used as a new molecular marker for the diagnosis and prognosis of ESCC, and the restoration of partially exhausted CD8^+^ T cells by inhibiting CD39 may be a new strategy for treating ESCC.

## 1. Introduction

According to the Global Cancer Statistics 2020 (GLOBOCAN 2020), esophageal carcinoma has the seventh highest number of cases and sixth highest number of deaths among the most common cancers in the world [1] and is a malignant tumor that seriously endangers human health; its main pathological type is esophageal squamous cell carcinoma (ESCC). Esophageal carcinoma has an insidious onset and a poor prognosis, and the overall 5-year survival rate of the patient is still less than 30% [2]. Therefore, identifying new molecular markers of prognosis and effective therapeutic drugs are crucial to prolonging the survival time of patients.

Ectonucleoside triphosphate diphosphohydrolase 1 (CD39) is encoded by the *ENTPD1* and exerts an immunosuppressive effect by the adenosine triphosphate (ATP)-adenosine (ADO) pathway [3]. Studies have shown that CD39 is highly expressed on several human tumors and is closely associated with several immune cells, especially CD8^+^ T cells, in the tumor microenvironment (TME) [4,5]. Moreover, it was reported that CD39 expression defines cell exhaustion in tumor-infiltrating CD8^+^ T cells (CD8^+^ TILs) [6], but CD39 could also be a useful marker of tumor-specific CD8^+^ TILs [7]. Furthermore, a study found that in ESCC the invasion of polymorphonuclear myeloid-derived suppressor cells (PMN-MDSCs) promotes CD39 expression on CD8^+^ T cells, decreases sensitivity to chemotherapy, and consequently leads to poor prognosis [8], suggesting a potential role of CD39 expression on CD8^+^ T cells in the progression of esophageal carcinoma. However, there are still relatively few studies on CD39-expressing CD8^+^ T cells in esophageal carcinoma.

Nomograms are quantitative analysis diagrams, and they have been used for prognosis prediction in a variety of cancers [9,10,11], having demonstrated predictive power superior to that of traditional single indicators. In this study, we established a nomogram model by integrating clinical indicators and CD39-expressing CD8^+^ T cells to more accurately predict the prognosis of patients with ESCC and further explored the potential of CD39-expressing CD8^+^ T cells as a therapeutic target of esophageal carcinoma by investigating the relationship between the expression of *ENTPD1* and inhibitory immune checkpoint molecules in the TME.

## 2. Materials and Methods

### 2.1. Patient Information

Surgical specimens and case information of ESCC patients who underwent surgery between 1 January 2009 and 31 December 2010 were collected. Inclusion criteria included: (1) clear pathological diagnosis, (2) no residual tumor cells at the microscopic surgical margins, (3) complete documentation of case data, (4) complete follow-up information. Exclusion criteria included: (1) esophageal adenocarcinoma (ESA), (2) combination of other malignant tumors or combination of other systemic diseases, such as immune system diseases, chronic infections, and infectious diseases. The clinical staging was based on the 8th edition of the Union for International Cancer Control (UICC)/American Joint Committee on Cancer (AJCC) [12]. The ending date of follow-up was 31 July 2015. A total of 95 individuals who met the criteria were finally enrolled, and 95 cancer tissue specimens and 71 paracancerous tissue specimens were obtained. The median time of follow-up for surviving patients was 69 months (55–78 months).

### 2.2. Multiplex Immunohistochemistry (mIHC) Test

For mIHC staining in this study, the same primary antibodies CD39, CD8, and cytokeratin (CK) were used, as well as the Opal Manual IHC kit (NEL801001KT). All specimens were scanned and imaged using TissueFAXS software (TissueGnostics, Vienna, Austria). Shanghai Outdo Biotech Company provided the TMA chip and completed the scanning imaging. For more information on ESCC tissues samples, please see Table S1 in the Supplementary Material.

### 2.3. Random Grouping and Determination of Optimal Cut-Off Values

Random grouping was performed using the car and survival packages in the R (version 4.2.1, https://www.r-project.org/, accessed on 10 October 2022) software, with the ratio of the training and validation groups set at 7:3. The X-tile (version 3.6.1, Yale University) software [13] was used in the training group to determine the optimal cut-off values for survival analysis of quantitative variables such as age, proportion of positive lymph nodes, and CD39 expression, and to classify these quantitative variables into low and high groups based on the optimal cut-off points.

### 2.4. Construction and Validation of the Nomogram

The nomogram of the training group was constructed using the rms package in R software. In addition, in the training group, we plotted the area under the curves (AUCs) of receiver operating characteristics (ROC) including nomograms and other independent prognostic factors of ESCC at 1, 3, and 5 years to assess the superiority of the nomogram model. Then, we evaluated the predictive performance of the model by the internal validation. In the training group, the calibration curves at 1, 3, and 5 years were used to evaluate the accuracy of the model; the concordance index (C-index) and AUCs at 1, 3, and 5 years were applied to assess the differentiation of the model; and the decision curve analyses (DCAs) at 1, 3, and 5 years were performed to assess the clinical usefulness of the model. To further validate the predictive ability of the nomogram model, in the validation group, the calibration curves, C-index, AUCs at 1, 3, and 5 years, and DCAs at 1, 3, and 5 years were also performed.

### 2.5. Risk Stratification

The nomogramFormula package in R software was used to calculate the risk score for each patient in the training group based on the nomogram model. The X-tile tool was then used to determine the optimal cut-off value and to classify these patients into low-risk and high-risk groups. Finally, a nomogram model for risk stratification was constructed and AUCs were calculated at 1, 3, and 5 years.

### 2.6. Differential Gene Expression Analysis

Differential gene expression analysis was based on the GSE33426 dataset (including 41 ESCC and 12 normal tissues samples) in the Gene Expression Omnibus (GEO) [14] and performed by the NetworkAnalyst platform with limma method [15].

### 2.7. Immune Infiltration in the TME

The CIBERSORT package in R software was used to compare the differences in the infiltration abundance of 22 immune cells (including CD8^+^ T cells) between these ESCC and normal samples and between high and low *ENTPD1*-expresing groups, respectively. Furthermore, the TISIDB [16], TIMER [17,18], and GEPIA2 [19] databases were used to analyze the relationship between *ENTPD1* and immune infiltration in the TME. Firstly, the correlation between *ENTPD1* expression levels in esophageal carcinoma and the abundance of tumor-infiltrating immune cells (TIICs) in the TME was explored using the TIMER and TISIDB databases; secondly, the association between the expression of *ENTPD1* and genetic markers of TIICs was investigated using the TIMER2.0 and GEPIA2 databases; finally, the TIMER2.0 and TISIDB databases were used to explore the relationship between the expression of *ENTPD1* and the currently effective inhibitory immune checkpoint molecules programmed cell death protein 1 (*PDCD1*/*PD-1*), cytotoxic T-lymphocyte-associated protein 4 (*CTLA4*), and hepatitis A virus cellular receptor 2 (*HAVCR2*/*TIM-3*) in esophageal carcinoma.

### 2.8. Statistical Analysis

Paired *t*-test or Wilcoxon signed-rank test were performed using IBM SPSS statistics (version 21, IBM Corp) on 71 pairs of cancer and paracancerous tissues to compare the differences in CD39 expression, CD39-expressing CD8^+^ T cells, and other clinical indicators between the two groups. Statistical differences were compared between the training group and validation group for each variable in R software using the chi-square test or Fisher’s exact test. Kaplan–Meier survival curves were plotted using the log-rank test, and a two-sided *p* < 0.05 was considered statistically significant. Univariate Cox regression analysis was used if the *p* > 0.05 of proportional hazards (PH) assumption; otherwise, the time-dependent Cox regression analysis was used. Variables with *p* < 0.10 of Cox regression analysis were tested for multicollinearity. Then, variables with a variance inflation factor (VIF) less than 5 (no multicollinearity) [20] were further analyzed using the multivariate Cox regression analysis, and the variable with a final *p* < 0.05 was considered an independent prognostic factor for ESCC. These variables were used to construct the nomogram model. Student’s two-sample *t*-test was used to compare the differences in the infiltration levels of 22 immune cells, and Spearman’s rank correlation was performed to assess the correlation between *ENTPD1* expression and immune infiltration of TME in the databases. GraphPad Prism (version 8.2, GraphPad Software, San Diego, CA, USA) and Adobe Illustrator (version CS6, Adobe Systems Incorporated, Mountain View, MA, USA) were used to generate and process the figures.

## 3. Results

### 3.1. Differences in the Expression of CD39 in ESCC Cancer and Paracancerous Tissues

**Figure 1:**
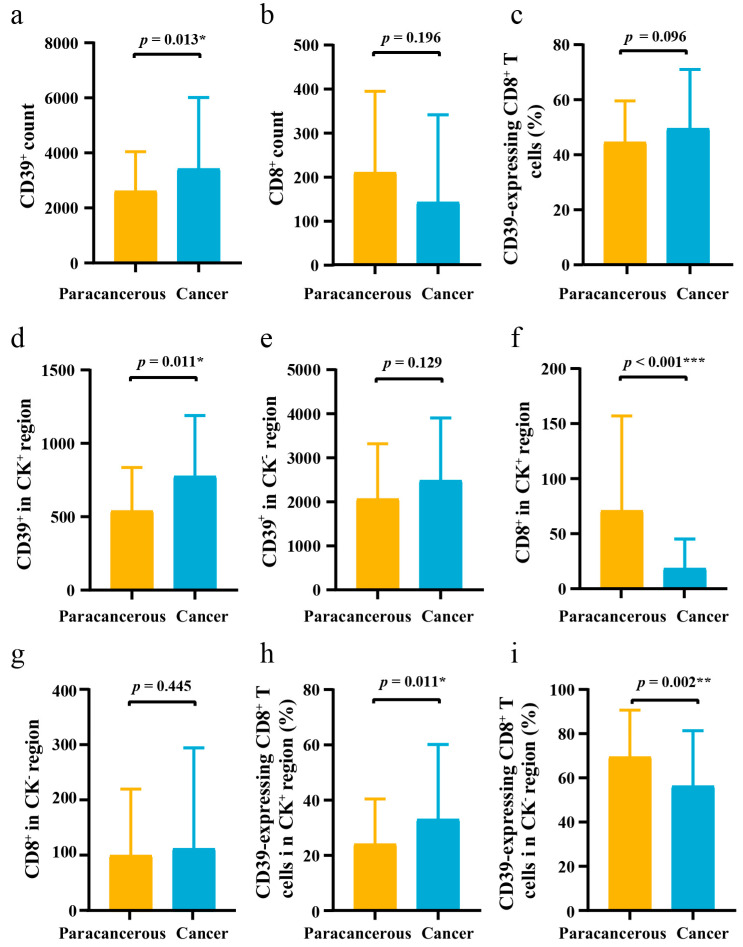
The difference of CD39 expression in 71 pairs of ESCC cancer tissues and paracancerous tissues. (**a**–**c**) The differences of CD39^+^ count, CD8^+^ count, and CD39-expressing CD8^+^ T cells between ESCC cancer tissues and paracancerous tissues; (**d**–**f**) The differences of CD39^+^ in CK^+^ region, CD39^+^ in CK^−^ region, and CD8^+^ in CK^+^ region between ESCC cancer tissues and paracancerous tissues; (**g**–**i**) The differences of CD8^+^ in CK^−^ region, CD39-expressing CD8^+^ T cells in CK^+^ region, and CD39-expressing CD8^+^ T cells in CK^−^ region between ESCC cancer tissues and paracancerous tissues. CD39, ectonucleoside triphosphate diphosphohydrolase 1; CK, cytokeratin; ESCC, esophageal squamous cell carcinoma; IQR, interquartile range; SD, standard deviation. The error bar represents median and interquartile range (IQR) in (**a**,**b**,**d**–**g**). The error bar represents mean and standard deviation (SD) in (**c**,**h**,**i**). * *p* < 0.05, ** *p* < 0.01, *** *p* < 0.001.

As shown in Figure 1 and Table S2, the expression level of CD39 was higher in ESCC cancer tissues than in paracancerous tissues (*p* = 0.013) and also in the CK^+^ region (*p* = 0.011). In addition, in the CK^+^ region, the levels of CD8^+^ (*p* < 0.001) were lower in cancer tissues, but the expression of CD39 on CD8^+^ T cells was higher (*p* = 0.011) in cancer tissues.

### 3.2. Clinical Characteristics of 95 ESCC Patients

Of the 95 included ESCC patients, 67 patients were eventually included in the training group and 28 patients in the validation group. Table 1 presents the clinical characteristics of patients in the training and validation groups. The results showed that there were no significant differences (*p* > 0.05) in all variables between the training and validation groups, and all variables were available for subsequent analysis.

### 3.3. Optimal Cut-Off Values and Cox Regression Analysis of the Training Group

**Figure 2:**
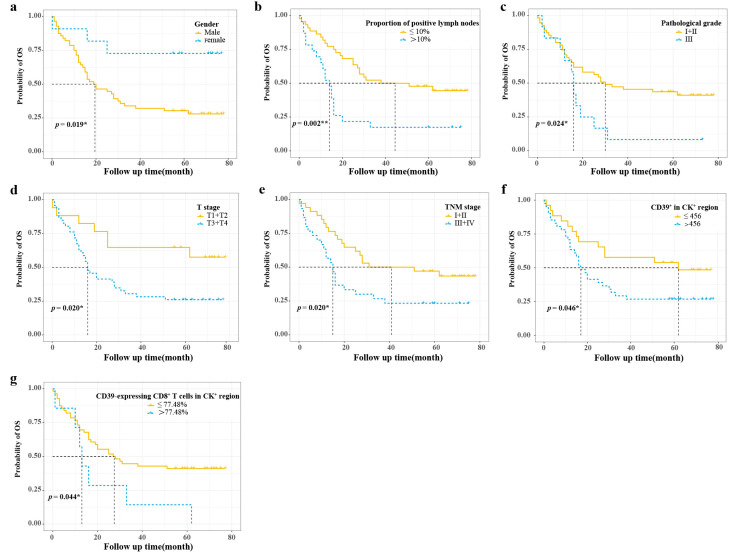
Survival curve analysis of prognostic variables with statistically significant differences. (**a**–**d**) Survival curve analyses of gender, proportion of positive lymph nodes, pathological grade, and T stage; (**e**–**g**) Survival curve analyses of TNM stage, CD39^+^ in CK^+^ region, and CD39-expressing CD8^+^ T cells in CK^+^ region. CD39, ectonucleoside triphosphate diphosphohydrolase 1; CK, cytokeratin; OS, overall survival. * *p* < 0.05, ** *p* < 0.01.

**Figure 3:**
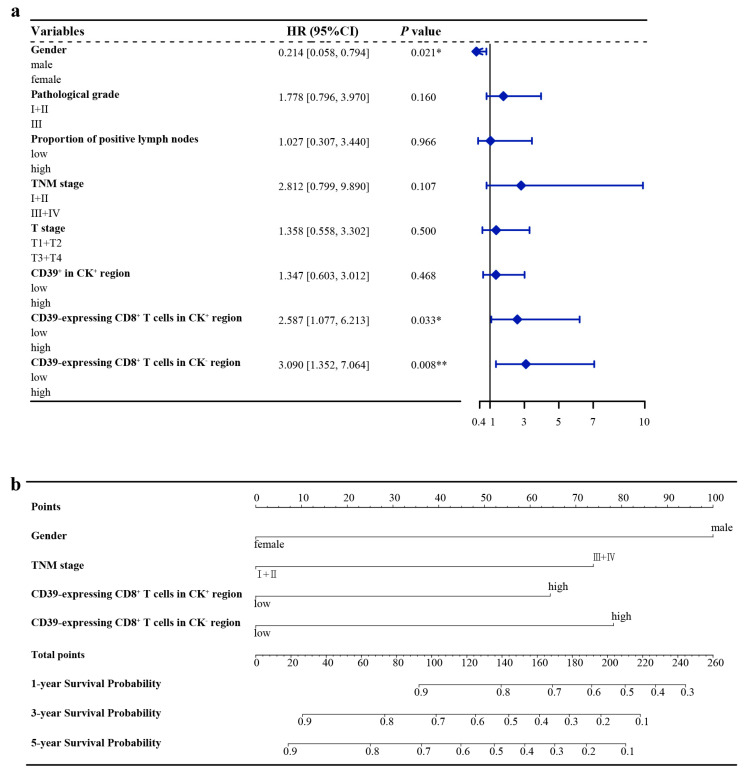
Hazard ratio of ESCC in the training group and nomogram model for OS of ESCC. (**a**) Hazard ratio of ESCC in the training group; (**b**) Nomogram model for OS of ESCC. CD39, ectonucleoside triphosphate diphosphohydrolase 1; CI, confidence interval; CK, cytokeratin; ESCC, esophageal squamous cell carcinoma; HR, hazard ratio; OS, overall survival. * *p* < 0.05, ** *p* < 0.01.

The optimal cut-off values calculated by X-tile software are shown in Table 2. The Kaplan–Meier survival curves for the statistically significant variables screened by the log-rank test are shown in Figure 2. Univariate Cox regression analysis showed that gender, proportion of positive lymph nodes, pathological grade, T stage, TNM stage, CD39^+^ in the CK^+^ region, CD39-expressing CD8^+^ T cells in the CK^+^ region, and CD39-expressing CD8^+^ T cells in the CK^−^ region were statistically different in the overall survival (OS). The PH assumptions of these variables were all greater than 0.05, and there was no multicollinearity (VIFs were all less than 5) (Table 2). Additionally, the ratio of the sample to the number of included variables was close to 10, which could be approximated as appropriate. Then, these variables were further included in the multivariate Cox regression analysis (Table 2). Finally, multivariate Cox regression analysis showed gender (HR, 0.214; 95% CI, 0.058–0.794; *p* = 0.021), CD39-expressing CD8^+^ T cells in the CK^+^ region (HR, 2.587; 95% CI, 1.077–6.213; *p* = 0.033), and CD39-expressing CD8^+^ T cells in the CK^−^ region (HR, 3.090; 95% CI, 1.352–7.064; *p* = 0.008) as independent prognostic factors for ESCC (Table 2) (Figure 3a).

### 3.4. Nomogram Construction

**Figure 4:**
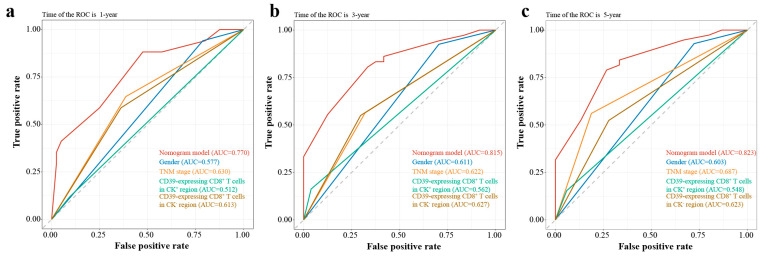
The AUCs at 1, 3, and 5 years of nomogram model and other independent prognostic factors of ESCC. (**a**) The 1-year AUCs of nomogram model and other independent prognostic factors of ESCC; (**b**) The 3-year AUCs of nomogram model and other independent prognostic factors of ESCC; (**c**) The 5-year AUCs of nomogram model and other independent prognostic factors of ESCC. AUC, area under the curve of receiver operating characteristics (ROC); CD39, ectonucleoside triphosphate diphosphohydrolase 1; CK, cytokeratin; ESCC, esophageal squamous cell carcinoma.

The variables screened by multivariate Cox regression were integrated into the construction of the nomogram model for the training group. In addition, TNM stage, a recognized prognostic factor for ESCC, was included in the nomogram model (Figure 3b). Details of the labels of the scales and points in the nomogram can be found in Table S3. Furthermore, the 1-year, 3-year, and 5-year AUCs of the nomogram model were higher than those of other independent prognostic factors of ESCC (Figure 4).

### 3.5. Nomogram Validation

**Figure 5:**
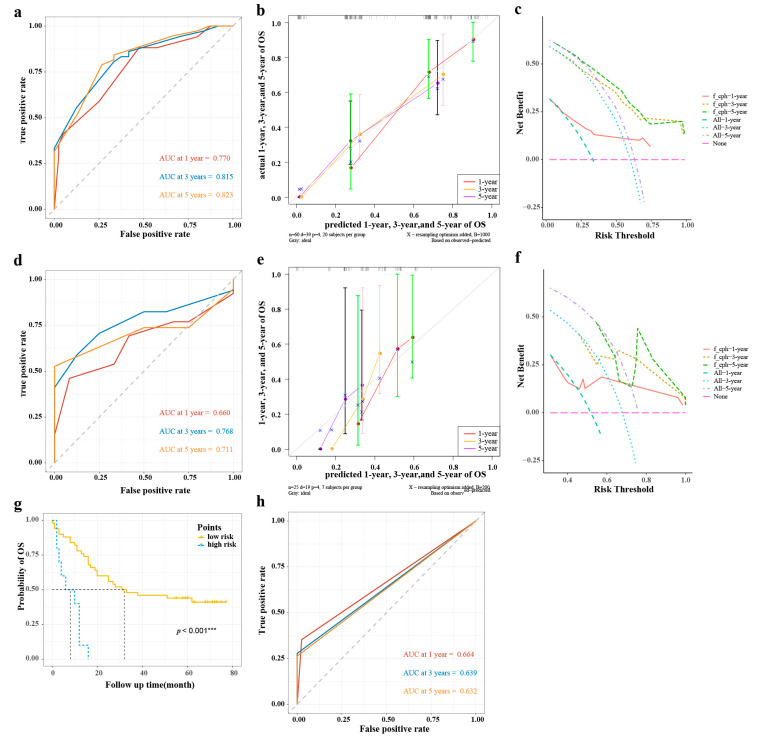
The validations of nomogram model and risk stratification of ESCC. (**a**,**d**) The AUCs at 1, 3, and 5 years of the training group and validation group; (**b**,**e**) The calibration curves at 1, 3, and 5 years of the training group and validation group; (**c**,**f**) The DCAs of the training group and validation group; (**g**) Survival curve analysis of risk stratification; (**h**) The AUCs at 1, 3, and 5 years of risk stratification in the training group. AUC, area under the curve of receiver operating characteristics (ROC); DCA, decision curve analysis; ESCC, esophageal squamous cell carcinoma; OS, overall survival. *** *p* < 0.001.

In the training group, the 1-year, 3-year, and 5-year AUCs were 0.770, 0815, and 0.823 (Figure 5a), respectively, and the C-index was 0.711. The calibration curves showed good consistency between predicted probabilities of OS at 1, 3, and 5 years in the nomogram and the actual observed probabilities (Figure 5b), and the DVA curves showed that the nomogram provided more benefit for ESCC patients compared with treatment/no treatment for predicting the probabilities of OS at 1, 3, and 5 years (Figure 5c).

In the validation group, the 1-year, 3-year, and 5-year AUCs were 0.660, 0.768, and 0.711, respectively (Figure 5d), and the C-index was 0.601. The calibration curves showed that the predicted probabilities of OS at 1, 3, and 5 years in the nomogram were in good agreement between the predicted outcome and the actual observation (Figure 5e), and the DVA curves also showed that the nomogram model provided more benefit for ESCC patients (Figure 5f).

### 3.6. Risk Stratification

As shown in Figure 5g, there was a significant difference in Kaplan–Meier survival curves for OS probabilities between the low-risk group (risk score ≤ 238) and high-risk group (risk score > 238) (*p* < 0. 001). Meanwhile, the AUCs at 1, 3, and 5 years were 0.664, 0.639, and 0.632, respectively (Figure 5h).

### 3.7. Differential Expression of ENTPD1 between ESCC and Normal Tissues

**Figure 6:**
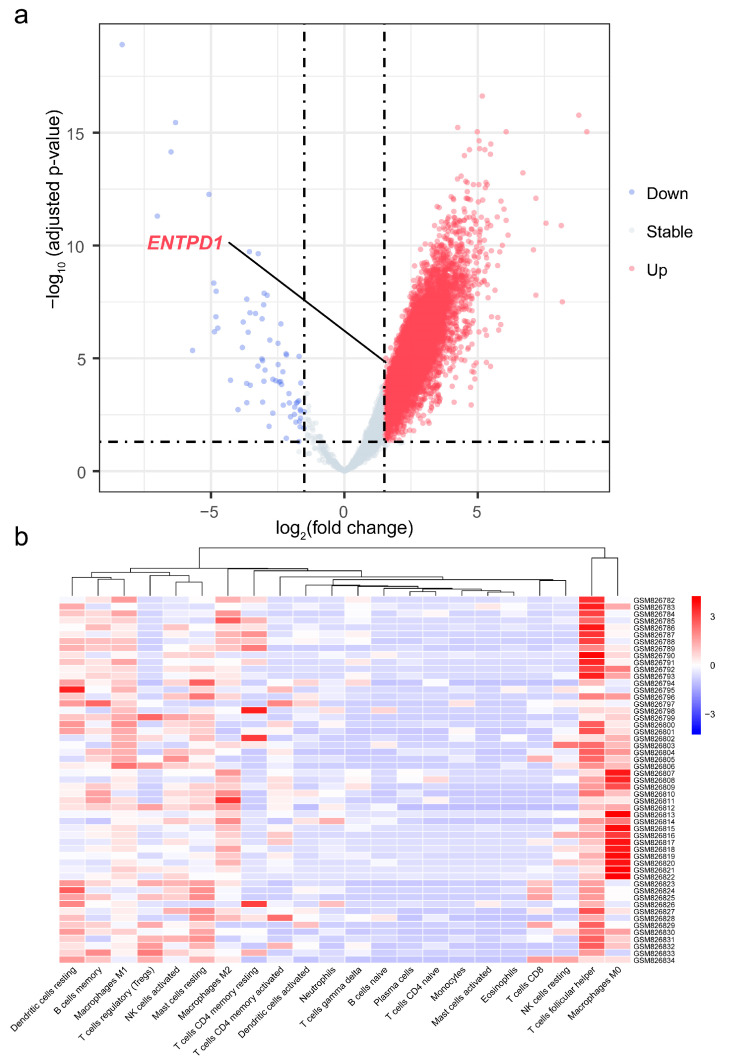
Volcano plot of differential gene expression analysis and heatmap of infiltration abundance of 22 immune cells. (**a**) Volcano plot of differential gene expression analysis of 41 ESCC and 12 normal tissues samples; (**b**) Heatmap of infiltration abundance of 22 immune cells of 41 ESCC and 12 normal tissues samples. *ENTPD1*, ectonucleoside triphosphate diphosphohydrolase 1.

**Figure 7:**
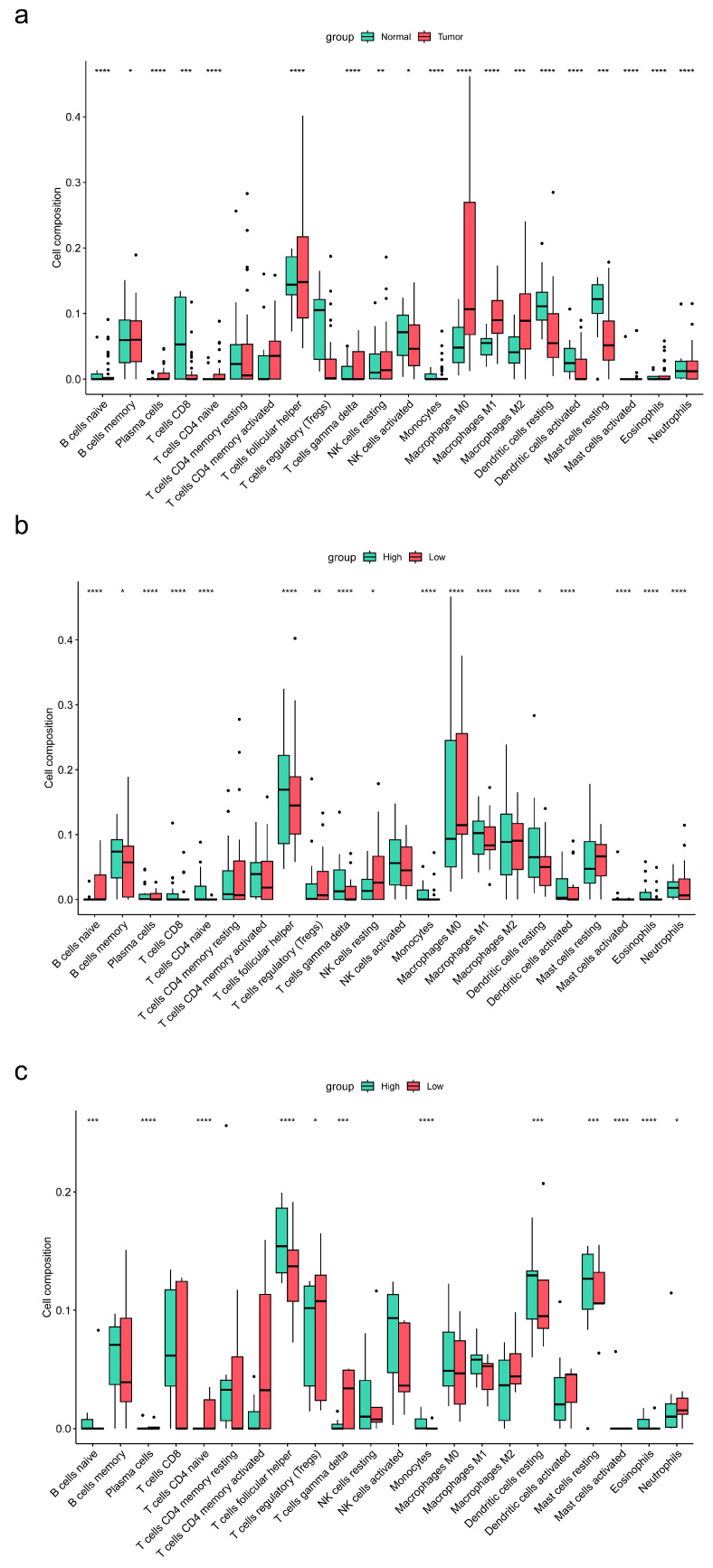
Box plots of the differences in the infiltration abundance of 22 immune cells between ESCC and normal samples and between high and low *ENTPD1*-expresing groups. (**a**) Box plot of the differences in the infiltration abundance of 22 immune cells between ESCC and normal samples; (**b**) Box plot of the differences in the infiltration abundance of 22 immune cells between high and low *ENTPD1*-expresing groups in ESCC samples; (**c**) Box plot of the differences in the infiltration abundance of 22 immune cells between high and low *ENTPD1*-expresing groups in normal samples. *ENTPD1*, ectonucleoside triphosphate diphosphohydrolase 1; ESCC, esophageal squamous cell carcinoma. * *p* < 0.05, ** *p* < 0.01, *** *p* < 0.001, **** *p* < 0.0001.

The volcano plot (Figure 6a) showed that the expression of *ENTPD1* was upregulated in ESCC tissues compared to normal tissues (adjusted *p* < 0.001; log_2_ fold change = 1.99), and the heatmap (Figure 6b) demonstrated the infiltration abundance of 22 immune cells between samples. Moreover, the box plot (Figure 7a) showed that the infiltration abundance of CD8^+^ T cells in ESCC tissues was lower than that in normal tissues (*p* < 0.001). In addition, the infiltration abundance of CD8^+^ T cells was significantly higher in the high *ENTPD1*-expressing group than in the low *ENTPD1*-expressing group in ESCC tissues (*p* < 0.0001) (Figure 7b); however, there was no significant difference in normal tissues (*p* > 0.05) (Figure 7c).

### 3.8. Association of ENTPD1 Expression with Immune Infiltration in TME of Esophageal Carcinoma

The results of the TISIDB database showed that *ENTPD1* expression was significantly positively correlated with the abundance of TIICs in the TME of most types of tumors, including esophageal carcinoma (Figure S1a). Furthermore, *ENTPD1* expression in the esophageal carcinoma was found to be positively correlated with both subtypes of CD8^+^ T cells: active CD8^+^ T cells (*p* < 0.001) and memory CD8^+^ T cells (*p* < 0.001) (Figure S1b,c). The TIMER database also showed consistent results that *ENTPD1* expression was significantly positively correlated with the infiltration abundance of CD8^+^ T cells in esophageal carcinoma (*p* = 0.028) (Figure S1d).

Furthermore, *ENTPD1* expression in esophageal carcinoma tissues was positively correlated with the expression of genetic markers of multiple TIICs, including *CD8A* and *CD8B* of CD8^+^ T cells, with or without adjustment for tumor purity (Table S4). The GEPIA2 database showed similar results; however, in normal tissues, there was no statistical correlation between the expression of *ENTPD1* and *CD8A*/*B* (Table S5).

In the TISIDB database, *ENTPD1* expression in esophageal carcinoma was shown to be statistically positively correlated with the expression of currently recognized inhibitory immune checkpoint molecules *PDCD1* (*p* < 0.001), *CTLA4* (*p* < 0.001), and *HAVCR2* (*p* < 0.001) (Figure S2a–c). Additionally, similar results were shown in the TIMER 2.0 database, and the *p*-values were all less than 0.001 (Figure S2d–f).

## 4. Discussion

Esophageal carcinoma is a highly aggressive and fatal malignancy with a poor prognosis in which the 5-year survival rate is less than 30%. It has become one of the major public health problems worldwide. CD39 has been found to be expressed at high levels in a variety of human tumors, including breast and ovarian cancers [5,21]; however, it has not been reported in esophageal carcinoma. Our study showed that CD39 was also highly expressed in ESCC cancer tissues and was further localized to the epithelial region as well. Furthermore, we found that high CD39-expressing CD8^+^ T cells, both in epithelial and non-epithelial regions, were independent risk factors for prognosis in ESCC patients. Furthermore, the expression of *ENTPD1* was also upregulated in ESCC tissues compared to normal tissues, demonstrating an action direction consistent with that of CD39 protein. These facts emphasize the unique role of CD39 in the progression of ESCC. What is more, we found that the infiltration abundance of CD8^+^ T cells was significantly higher in the high *ENTPD1*-expressing group than in the low *ENTPD1*-expressing group in ESCC tissues. Moreover, we found that the expression level of *ENTPD1* was positively correlated with the infiltrating abundance of CD8^+^ T cells and the expression level of inhibitory immune checkpoint molecules *PDCD1*, *CTLA4*, and *HAVCR2* in esophageal carcinoma, but not in normal tissues. These facts suggest that the expression of CD39 most likely defines highly exhausted CD8^+^ T cells in ESCC which are unable to exert anti-tumor effects, and CD39-expressing CD8^+^ T cells could as a new molecular marker for diagnosis and prognosis of ESCC.

In some cases, tumor cells themselves overexpress CD39 in TME compared to normal cells, but the major cell types that express CD39 most stably are immune cells and certain subsets of stromal cells, including B cells, CD8^+^ T cells, and NK cells. Initially, Gupta et al. [22] identified CD39 as a marker of exhausted CD8^+^ T cells. They observed that pathogen-specific CD8^+^ T cells against HCV or HIV expressed high levels of CD39, and they further found that these CD39 were enzymatically active and co-expressed with PD-1, with a transcriptional signature of T cell exhaustion. Later, Canale et al. [6] found that CD39^+^CD8^+^ T cells from breast cancer and melanoma patients were characterized by intra-tumor exhaustion and invasive/metastatic lymph nodes and demonstrated that CD8^+^ T cells highly expressed CD39 and strongly hydrolyzed extracellular ATP by establishing a mouse tumor model. This feature highlights the potential of exhausted CD8^+^ T cells to contribute to the suppressive TME by regulating the ATP-adenosine diphosphate (ADP) balance. Similarly, Thelen et al. [23] found CD39^+^CD8^+^ T cell accumulation in head and neck squamous cell carcinoma, renal cell carcinoma, non-small cell lung cancer, and gastric adenocarcinoma. By using RNA-Sequencing, Simoni et al. [7] also found that CD39^+^CD8^+^ T cells exhibited exhaustion characteristics in lung cancer and CRC. Additionally, Qi et al. [24] observed that high numbers of CD39^+^CD8^+^ T cells indicated poor prognosis in clear cell renal cell carcinoma. Together, these studies coincidentally present the view that CD39 suppresses the anti-tumor response by exhausting CD8^+^ T cells and is associated with poor prognosis of cancer. Similarly, we observed that CD39 is overexpressed, especially on the CD8^+^ T cells in ESCC cancer tissues, and the high CD39-expressing CD8^+^ T cells in the CK^+^ and CK^−^ regions were independent predictors of poor prognosis, which was consistent with the above review. Puzzlingly, in our study, CD39-expressing CD8^+^ T cells in the CK^−^ region were lower in cancer tissues compared to paracancerous tissues, which contradicted the previous view. Nevertheless, CK^+^ is localized in the epithelial region, where tumor cells accumulate; CD39-expressing CD8^+^ T cells in the CK^+^ region are relatively more valuable for the diagnosis of ESCC. In short, high CD39-expressing CD8^+^ T cells can be used as a diagnostic and prognostic predictor of ESCC.

Furthermore, in vivo and in vitro data have suggested that CD39 antibodies in combination with PD-1/PD-L1 inhibitors may improve anti-tumor efficacy [25]. Overall, this evidence seems to indirectly present the exhaustion characteristic of high CD39-expressing CD8^+^ T cells and further shows its potential as a target in combination with ICIs for the treatment of esophageal carcinoma. Interestingly, a study [8] found that in ESCC, CD39 expression on CD8^+^ T cells was elevated by the invasion of PMN-MDSCs, which played an immunosuppressive role. However, it remains unclear whether PMN-MDSCs or other factors regulate exhausted CD39^+^CD8^+^ T cells and whether these exhausted cells exert immunosuppressive effects by the ATP-ADO or other pathways in ESCC. Additionally, CD39-expressing CD8^+^ T cells showed regulatory properties. The results of Gallerano et al. [26] identified for the first time a single nucleotide polymorphism (SNP rs10748643 A > G) as a genetic factor for CD39 expression in CD8^+^ T cells in CRC patients and demonstrated the inhibitory effect of CD39^+^CD8^+^ T cells in vitro. An increased effector function of CD39^+^CD8^+^ T cells was further observed by inhibiting CD39-associated ATPase. These data undoubtedly reinforce the view genetically that CD39^+^CD8^+^ T cells are most likely a suitable diagnostic indicator for cancer including ESCC and further provide a novel strategy for restoring partially exhausted CD8^+^ T cells or suppressing CD8^+^ T cells with regulatory properties by inhibiting CD39.

Notably, studies have also found a potential role of CD39^+^CD8^+^ T in monitoring immune checkpoint therapy. The poorer response of epidermal growth factor receptor (EGFR)-mutant lung cancer to anti-PD-1 therapy may be related to the lower abundance of CD39^+^CD8^+^ T cells [7]. Additionally, some studies have described CD39^+^CD8^+^ T cells as tumor antigen-specific responsive cells [27,28] and further attributed most of these multifunctional and protective effects to CD103 [29]. However, the current evidence on this remains insufficient. Overall, CD39 is driven by tumor antigens and overexpressed on exhausted CD8^+^ T cells. At the same time, CD39^+^CD8^+^ T cells show a broad prospect as a biomarker for diagnosis and prognosis prediction of cancer, as well as an immunotherapeutic target. This also is reflected in the results of our study on ESCC.

Compared with other clinical prediction models, the nomogram model is more intuitive and accurate in predicting the survival probability of each patient and plays an increasingly important role in clinical prognosis assessment [9,30]. In this study, we developed a nomogram model for predicting the probability of OS in ESCC patients based on gender, TNM stage, CD39-expressing CD8^+^ T cells in the CK^+^ region, and CD39-expressing CD8^+^ T cells in the CK^−^ region. Moreover, the C-indexes, AUCs, calibration curves, and DCA curves at 1, 3, and 5 years for both the training and validation groups all showed that the nomogram model provided a more accurate and reliable prognostic prediction for ESCC patients. On this basis, we further risk-stratified ESCC patients according to their scores, and Kaplan–Meier survival curves also showed significant differences in OS between high- and low-risk groups. The 1-year, 3-year, and 5-year survival of AUCs verified the good accuracy of the risk stratification model. This could provide a reference for clinicians to develop more appropriate individualized treatment plans for patients with different risk score rankings so as to further improve prognosis.

The use of mIHC technology is an advantage of this study, as it allows the simultaneous detection of multiple markers from a single tissue sample and is promising in the field of cancer immunology [31]. In addition, we assessed the effects of CD39-expressing CD8^+^ T cells on the progression of ESCC at the protein and RNA levels, respectively, resulting in more comprehensive and reliable results. Certainly, there are certain limitations of our study. The single-center study design and small sample size may lead to potential bias in the results. In the future, large epidemiological studies with multicenter design are strongly needed to provide more clinical data to validate our results, especially randomized controlled trials (RCTs).

## 5. Conclusions

In conclusion, the CD39-expressing CD8^+^ T cells can be used as a diagnostic and prognostic predictor for patients with ESCC. Meanwhile, the nomogram model combing CD39-expressing CD8^+^ T cells with clinical indicators including gender and TNM stage can better predict the prognosis of ESCC patients, and further risk stratification can provide a basis for clinicians to develop individualized treatment plans. Furthermore, restoration of partially exhausted CD8^+^ T cells by inhibiting CD39 may be a new strategy for treating ESCC.

## Figures and Tables

**Table 1:** Clinical characteristics of 95 ESCC patients.

Variables	Training Group	Validation Group	*p*-Value ^a^
Number	67	28	
Age, mean ± SD, months	65.91 ± 9.12	66.43 ± 8.23	0.79
Tumor length, mean ± SD, cm	4.66 ± 1.79	5.06 ± 1.74	0.22
Tumor volume, mean ± SD, cm^3^	21.18 ± 17.91	26.63 ± 27.16	0.52
Proportion of positive lymph nodes, mean ± SD, %	15.68 ± 24.61	13.93 ± 16.17	0.46
CD39^+^ count, mean ± SD	4014.48 ± 2957.20	3559.89 ± 2393.59	0.68
CD8^+^ count, mean ± SD	277.79 ± 418.32	278.93 ± 395.91	0.32
CD39-expressing CD8^+^ T cells, mean ± SD, %	50.53 ± 21.50	46.70 ± 21.83	0.44
CD39^+^ in CK^+^ region, mean ± SD	831.16 ± 753.49	1191.30 ± 1201.52	0.20
CD39^+^ in CK^−^ region, mean ± SD	3183.31 ± 2786.14	2368.59 ± 1707.59	0.35
CD8^+^ in CK^+^ region, mean ± SD	45.93 ± 101.88	79.81 ± 200.42	0.44
CD8^+^ in CK^−^ region, mean ± SD	231.87 ± 335.91	199.11 ± 231.87	0.49
CD39-expressing CD8^+^ T cells in CK^+^ region, mean ± SD, %	36.06 ± 28.32	26.21 ± 25.31	0.11
CD39-expressing CD8^+^ T cells in CK^−^ region, mean ± SD, %	56.58 ± 24.80	55.14 ± 28.05	0.95
Gender, *n* (%)			0.49
male	56 (83.60)	21 (75.00)	
female	11 (16.40)	7 (25.00)	
Pathological grade, *n* (%)			1.00
I + II	55 (82.10)	23 (82.10)	
III	12 (17.90)	5 (17.90)	
Vascular invasion, *n* (%)			0.69
no	62 (92.50)	25 (89.30)	
yes	5 (7.50)	3 (10.70)	
T stage, *n* (%)			0.68
T1 + T2	17 (27.00)	5 (20.00)	
T3 + T4	46 (73.00)	20 (80.00)	
TNM stage, *n* (%)			0.49
I + II	34 (53.10)	11 (42.30)	
III + IV	30 (46.90)	15 (57.70)	

CD39, ectonucleoside triphosphate diphosphohydrolase 1; CK, cytokeratin; ESCC, esophageal squamous cell carcinoma; SD, standard deviation. ^a^ Statistical differences were compared between the training group and validation group for each variable in R software using the chi-square test or Fisher’s exact test.

**Table 2:** Univariate and multivariate Cox regression analysis of 67 ESCC patients.

Variables	Cut-Off Value	PH	Univariate/Time-Dependent Cox Regression			Multicollinearity	Multivariate Cox Regression		
		*p*-Value	HR	95%CI	*p*-Value	VIF	HR	95%CI	*p*-Value
Gender		0.490				1.315			
male			1.000	Reference			1.000	Reference	
female			0.271	0.084–0.877	0.029 **		0.214	0.058–0.794	0.021 **
Age (years)	72	0.880							
≤72			1.000	Reference					
>72			0.676	0.333–1.373	0.279				
Tumor length (cm)	3	0.019 **							
≤3			1.000	Reference					
>3			1.078	0.752–1.546	0.680				
Tumor volume (cm^3^)	12	0.180							
≤12			1.000	Reference					
>12			0.605	0.329–1.112	0.106				
Proportion of positive lymph nodes	10%	0.390				3.025			
≤10%			1.000	Reference			1.000	Reference	
>10%			2.599	1.407–4.803	0.002 **		1.027	0.307–3.440	0.966
Pathological grade		0.100				1.142			
I + II			1.000	Reference			1.000	Reference	
III			2.215	1.100–4.460	0.026 **		1.778	0.796–3.970	0.160
Vascular invasion		0.890							
no			1.000	Reference					
yes			1.720	0.613–4.832	0.303				
T stage		0.820				1.336			
T1 + T2			1.000	Reference			1.000	Reference	
T3 + T4			2.554	1.126–5.793	0.025 **		1.358	0.558–3.302	0.500
TNM stage		0.260				2.778			
I + II			1.000	Reference			1.000	Reference	
III + IV			2.049	1.110–3.784	0.022 **		2.812	0.799–9.890	0.107
CD39^+^ count	2821	0.100							
≤2821			1.000	Reference					
>2821			1.616	0.879–2.969	0.122				
CD39-expressing CD8^+^ T cells	30.10%	0.610							
≤30.10%			1.000	Reference					
>30.10%			1.722	0.726–4.084	0.217				
CD39^+^ in CK^+^ region	456	0.980				1.321			
≤456			1.000	Reference			1.000	Reference	
>456			1.924	1.000–3.700	0.050 *		1.347	0.603–3.012	0.468
CD39^+^ in CK^−^ region	3248	0.880							
≤3248			1.000	Reference					
>3248			1.424	0.771–2.633	0.259				
CD39-expressing CD8^+^ T cells in CK^+^ region	77.48%	0.220				1.053			
≤77.48%			1.000	Reference			1.000	Reference	
>77.48%			2.282	1.004–5.190	0.049 **		2.587	1.077–6.213	0.033 **
CD39-expressing CD8^+^ T cells in CK^−^ region	59.46%	0.450				1.167			
≤59.46%			1.000	Reference			1.000	Reference	
>59.46%			1.738	0.953–3.170	0.071 *		3.090	1.352–7.064	0.008 **

95% CI, 95% confident interval; ESCC, esophageal squamous cell carcinoma; HR, hazard ratio; VIF, variance inflation factor. * p < 0.10, ** *p* < 0.05.

## Data Availability

The data that support the findings of our study are available from the corresponding authors upon reasonable request. The GSE33426 dataset in the Gene Expression Omnibus (GEO) can be found at: https://www.ncbi.nlm.nih.gov/geo/query/acc.cgi?acc=GSE33426 (accessed on 20 December 2022). The results of association of *ENTPD1* expression with immune infiltration in TME of esophageal carcinoma here can be found in the databases of TISIDB (http://cis.hku.hk/TISIDB/index.php, accessed on 15 April 2022), TIMER (https://cistrome.shinyapps.io/timer/, accessed on 15 April 2022), and GEPIA2 (http://gepia2.cancer-pku.cn, accessed on 15 April 2022).

## Supplementary Materials

The following supporting information can be downloaded at: https://www.mdpi.com/article/10.3390/cancers15041184/s1. Figure S1: Correlation of *ENTPD1* expression with the abundance of TIICs in ESCA; Figure S2: Correlation of *ENTPD1* expression with the expression of inhibitory immune checkpoint molecules in ESCA; Table S1: Detailed information of ESCC tissues samples; Table S2: The difference of CD39 expression in 71 pairs of ESCC cancer tissues and paracancerous tissues; Table S3: Points for variables in nomogram models; Table S4: Correlation of *ENTPD1* expression with the expression of genetic markers of ESCA TIICs in the TIMER2.0 database; Table S5: Correlation of *ENTPD1* expression with genetic markers of ESCA TIICs in the GEPIA2 database.
