# Supplementary material for: CD39-Expressing CD8+ T Cells as a New Molecular Marker for Diagnosis and Prognosis of Esophageal Squamous Cell Carcinoma
Source: Cancers (Basel). 2023 Feb 13;15(4):1184. doi: 10.3390/cancers15041184 (PMC9954671; doi:10.3390/cancers15041184)
Supplement: Supplementary file 1 [file cancers-15-01184-s001.zip › cancers-2172913-supplementary.pdf]

Supplementary Material

Table S1. Detailed information of ESCC tissues samples.

Items	Detailed information
Sources	The TMA chip containing these ESCC tissue samples was purchased from the Shanghai Outdo Biotech Company.
Procedure of mIHC experiment	1) Drying<br>2) Dewaxing<br>3) Antigen repair<br>4) Removal of endogenous peroxidase<br>5) Blocking<br>6) Primary antibody incubation<br>7) Incubation of secondary antibody<br>8) Opal dye color development<br>9) Microwave treatment<br>10) Repeat steps 5)-9) for subsequent indicators until all indicators are labeled.<br>11) DAPI staining and blocking<br>12) Photographic analysis
Interpretation of mIHC results	From the analysis report provided by Shanghai Outdo Biotech Company. The analysis report has provided the number of positive cells for each staining molecule, as well as the number of positive cells between two staining molecules. And it can be used directly for statistical analysis.

mIHC, multiplex immunohistochemistry.

Table S2. The difference of CD39 expression in 71 pairs of ESCC cancer tissues and paracancerous tissues.

Variables (Cancer tissue - paracancerous tissue)	Normality test					Paired t-test/Wilcoxon signed-rank test
	P value	Mean	SD	Median	IQR	*P* value
$CD39^+$ count	0.002**			1035.500	3560.500	0.013*
$CD8^+$ count	<0.001***			-45.000	358.750	0.196
CD39-expressing $CD8^+$ T cells	0.200	4.832	25.007			0.096
$CD39^+$ in CK^+ region	<0.001***			91.500	772.750	0.011*
$CD39^+$ in CK^- region	<0.001***			461.500	2605.750	0.129
$CD8^+$ in CK^+ region	<0.001***			-46.500	109.750	<0.001***
$CD8^+$ in CK^- region	<0.001***			10.500	272.250	0.445
CD39-expressing $CD8^+$ T cells in CK^+ region	0.200	9.155	28.179			0.011*
CD39-expressing $CD8^+$ T cells in CK^- region	0.200	-12.031	31.673			0.002**

CD39, extracellular nucleotide triphosphate diphosphate hydrolase 1; CK, cytokeratin; ESCC, esophageal squamous cell carcinoma; IQR, interquartile range; SD, standard deviation. *$P < 0.05$, **$P < 0.01$, ***$P < 0.001$.

Table S3. Points for variables in nomogram models.

Variables	Points	Total points	Nomogram		
			1-year	3-year	5-year
Gender		244	0.3		
male	100	227	0.4		
female	0	210	0.5		
TNM stage		191	0.6		
Ⅰ+Ⅱ	0	169	0.7		
Ⅲ+Ⅳ	74	139	0.8		
CD39-expressing $CD8^+$ T cells in CK^+ region		93	0.9		
≤77.48%	0	219		0.1	
>77.48%	64	196		0.2	
CD39-expressing $CD8^+$ T cells in CK^- region		178		0.3	
≤59.46%	0	161		0.4	
>59.46%	78	144		0.5	
		125		0.6	
		103		0.7	
		73		0.8	
		27		0.9	
		210			0.1
		188			0.2
		170			0.3
		153			0.4
		136			0.5
		117			0.6
		94			0.7
		65			0.8
		18			0.9

CD39, extracellular nucleotide triphosphate diphosphate hydrolase 1; CK, cytokeratin.

Table S4. Correlation of *ENTPD1* expression with the expression of genetic markers of ESCA TIICs in the TIMER2.0 database.

Immune cells	Genetic markers	Unadjusted tumor purity		Adjusted tumor purity	
		rho	*P* value [a]	rho	*P* value [a]
CD8+ T cell	*CD8A*	0.496	<0.001***	0.458	<0.001***
	CD8B	0.461	<0.001***	0.424	<0.001***
T cell (general)	*CD3D*	0.536	<0.001***	0.489	<0.001***
	CD3E	0.565	<0.001***	0.520	<0.001***
	CD2	0.607	<0.001***	0.570	<0.001***
B cell	*CD19*	0.417	<0.001***	0.355	<0.001***
	CD79A	0.437	<0.001***	0.389	<0.001***
Monocyte	*CD86*	0.502	<0.001***	0.458	<0.001***
	CD115 (CSF1R)	0.641	<0.001***	0.618	<0.001***
TAM	*CCL2*	0.529	<0.001***	0.491	<0.001***
	CD68	0.289	<0.001***	0.252	<0.001***
	IL10	0.534	<0.001***	0.503	<0.001***
M1 Macrophage	*INOS (NOS2)*	0.218	0.004**	0.241	0.002**
	IRF5	-0.081	0.303	-0.136	0.086
	COX2 (PTGS2)	0.282	<0.001***	0.257	<0.001***
M2 Macrophage	*CD163*	0.611	<0.001***	0.58	<0.001***
	VSIG4	0.515	<0.001***	0.48	<0.001***
	MS4A4A	0.617	<0.001***	0.585	<0.001***
Neutrophils	*CD66b (CEACAM8)*	0.125	0.104	0.106	0.184
	CD11b (ITGAM)	0.490	<0.001***	0.446	<0.001***
	CCR7	0.614	<0.001***	0.575	<0.001***
Natural killer cell	*KIR2DL1*	0.183	0.017*	0.136	0.095
	KIR2DL3	0.195	0.011*	0.179	0.025*
	KIR2DL4	0.204	0.008**	0.158	0.051
	KIR3DL1	0.274	<0.001***	0.231	0.003**
	KIR3DL2	0.131	0.093	0.075	0.371
	KIR3DL3	0.020	0.809	0.017	0.862
	KIR2DS4	0.123	0.118	0.106	0.202
Dendritic cell	*HLA-DPB1*	0.603	<0.001***	0.568	<0.001***
	HLA-DQB1	0.372	<0.001***	0.32	<0.001***
	HLA-DRA	0.572	<0.001***	0.536	<0.001***
	HLA-DPA1	0.610	<0.001***	0.581	<0.001***
	BDCA-1 (CD1C)	0.458	<0.001***	0.408	<0.001***
	BDCA-4 (NRP1)	0.595	<0.001***	0.565	<0.001***
	CD11c (ITGAX)	0.620	<0.001***	0.59	<0.001***
Th1	*T-bet (TBX21)*	0.516	<0.001***	0.465	<0.001***
	STAT4	0.666	<0.001***	0.635	<0.001***
	STAT1	0.296	<0.001***	0.252	<0.001***
	IFN (IFNG)	0.370	<0.001***	0.318	<0.001***

	TNF	0.211	0.006**	0.158	0.052
Th2	*GATA3*	0.379	<0.001***	0.33	<0.001***
	STAT6	0.235	0.002**	0.262	<0.001***
	STAT5A	0.565	<0.001***	0.542	<0.001***
	IL13	0.429	<0.001***	0.385	<0.001***
Tfh	*BCL6*	0.005	0.948	-0.009	0.923
	IL21	0.269	<0.001***	0.219	0.005**
Th17	*STAT3*	0.284	<0.001***	0.258	<0.001***
	IL17A	0.159	0.040*	0.164	0.040*
Treg	*FOXP3*	0.676	<0.001***	0.655	<0.001***
	CCR8	0.715	<0.001***	0.691	<0.001***
	STAT5B	0.530	<0.001***	0.556	<0.001***
	TGF (TGFB1)	-0.035	0.668	-0.114	0.160
T cell exhaustion	*PD-1 (PDCD1)*	0.539	<0.001***	0.503	<0.001***
	CTLA4	0.610	<0.001***	0.575	<0.001***
	LAG3	0.421	<0.001***	0.378	<0.001***
	TIM-3 (HAVCR2)	0.647	<0.001***	0.616	<0.001***
	GZMB	0.318	<0.001***	0.253	0.001**

ENTPD1, ectonucleoside triphosphate diphosphohydrolase 1; ESCA, esophageal carcinoma; TIICs, tumor-infiltrating immune cells; rho, correlation coefficient. **P*< 0.05, ***P*< 0.01, ****P*< 0.001. [a] Spearman's rank correlation was performed.

Table S5. Correlation of *ENTPD1* expression with genetic markers of ESCA TIICs in the GEPIA2 database.

Immune cells	Genetic markers	Tumor tissues		Normal tissues	
		rho	*P* value [a]	rho	*P* value [a]
CD8+ T-Cell	*CD8A*	0.490	<0.001***	0.290	0.340
	CD8B	0.460	<0.001***	0.320	0.280
Naive T-Cell	*CCR7*	0.600	<0.001***	0.550	0.055
	LEF1	0.300	<0.001***	0.280	0.350
	TCF7	0.230	0.002**	0.120	0.710
	SELL	0.660	<0.001***	0.530	0.064
Effector T-Cell	*CX3CR1*	0.520	<0.001***	0.740	0.005**
	FGFBP2	0.130	0.081	0.480	0.097
	FCGR3A	0.520	<0.001***	0.750	0.003**
Effector memory T-Cell	*PDCD1*	0.570	<0.001***	0.470	0.110
	DUSP4	0.290	<0.001***	-0.320	0.280
	GZMK	0.620	<0.001***	0.220	0.460
	GZMA	0.390	<0.001***	0.500	0.079
	IFNG	0.380	<0.001***	0.250	0.410
Central memory T-Cell	*CCR7*	0.600	<0.001***	0.550	0.055
	SELL	0.660	<0.001***	0.530	0.064
	IL7R	0.560	<0.001***	0.170	0.580
Resident memory T-Cell	*CD69*	0.640	<0.001***	0.700	0.008**
	ITGAE	0.033	0.660	0.520	0.074
	CXCR6	0.590	<0.001***	-0.110	0.710
	MYADM	0.500	<0.001***	0.750	0.004**
Exhausted T-Cell	*HAVCR2*	0.680	<0.001***	0.850	<0.001***
	TIGIT	0.600	<0.001***	0.430	0.140
	LAG3	0.400	<0.001***	0.760	0.004**
	PDCD1	0.570	<0.001***	0.470	0.110
	CXCL13	0.410	<0.001***	-0.071	0.820
	LAYN	0.320	<0.001***	0.820	<0.001***
Resting Treg T-Cell	*FOXP3*	0.690	<0.001***	0.210	0.490
	IL2RA	0.660	<0.001***	0.870	<0.001***
Effector Treg T-Cell	*FOXP3*	0.690	<0.001***	0.210	0.490
	CTLA4	0.630	<0.001***	0.200	0.520
	CCR8	0.720	<0.001***	-0.064	0.840
	TNFRSF9	0.580	<0.001***	0.390	0.180
Th1-like	*CXCL13*	0.410	<0.001***	-0.071	0.820
	HAVCR2	0.680	<0.001***	0.850	<0.001***
	IFNG	0.380	<0.001***	0.250	0.410
	CXCR3	0.500	<0.001***	0.470	0.110

	BHLHE40	-0.001	0.990	0.680	0.013*
	CD4	0.740	<0.001***	0.810	0.001**

ENTPD1, ectonucleoside triphosphate diphosphohydrolase 1; ESCA, esophageal carcinoma; TIICs, tumor-infiltrating immune cells; rho, correlation coefficient. $^{*}P < 0.05$, $^{**}P < 0.01$, $^{***}P < 0.001$. [a] Spearman's rank correlation was performed.

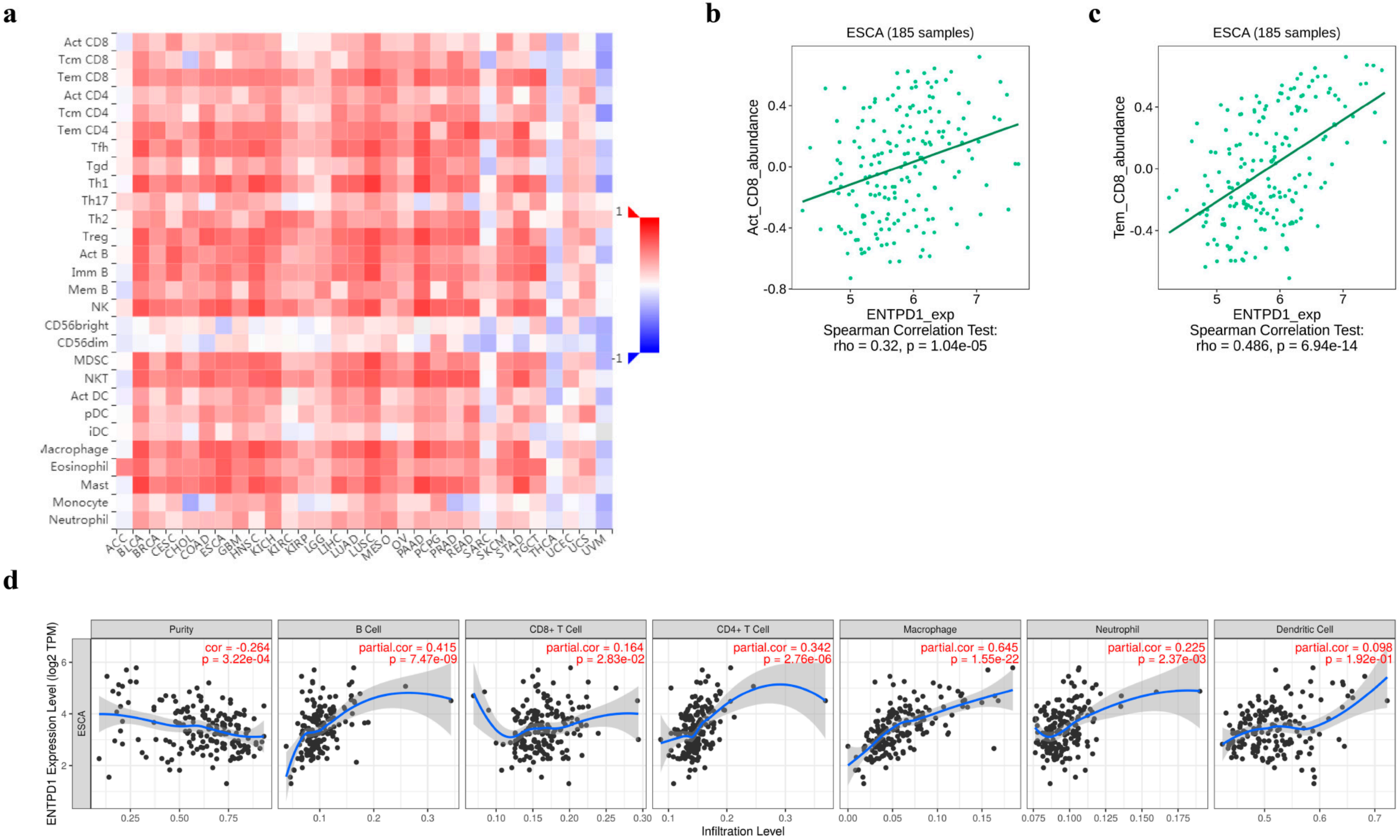
a
Act CD8
Tcm CD8
Tem CD8
Act CD4
Tcm CD4
Tem CD4
Tfh
Tgd
Th1
Th17
Th2
Treg
Act B
Imm B
Mem B
NK
CD56bright
CD56dim
MDSC
NKT
Act DC
pDC
iDC
Macrophage
Eosinophil
Mast
Monocyte
Neutrophil
ACC BLCA BRCA CESC CHOL COAD ESCA GBM HNSC KICH KIRC KIRP LGG LIHC LUAD LUSC MESO OV PAAD PCPG PRAD READ SARC SKCM STAD TGCT THCA UCEC UCS UVM
1
-1
b
ESCA (185 samples)
Act_CD8_abundance
ENTPD1_exp
Spearman Correlation Test:
rho = 0.32, p = 1.04e-05
c
ESCA (185 samples)
Tem_CD8_abundance
ENTPD1_exp
Spearman Correlation Test:
rho = 0.486, p = 6.94e-14
d
ENTPD1 Expression Level (log2 TPM)
ESCA
Purity
cor = -0.264
p = 3.22e-04
B Cell
partial.cor = 0.415
p = 7.47e-09
CD8+ T Cell
partial.cor = 0.164
p = 2.83e-02
CD4+ T Cell
partial.cor = 0.342
p = 2.76e-06
Macrophage
partial.cor = 0.645
p = 1.55e-22
Neutrophil
partial.cor = 0.225
p = 2.37e-03
Dendritic Cell
partial.cor = 0.098
p = 1.92e-01
Infiltration Level

Figure S1. Correlation of *ENTPD1* expression with the abundance of TIICs in ESCA [a]. (**a**) Correlation of *ENTPD1* expression with the abundance of TIICs in the TISIDB database; (**b, c**) Correlation of *ENTPD1* expression with the abundances of active $CD8^+$ T cells and memory $CD8^+$ T cell in the TISIDB database; (**d**) Correlation of *ENTPD1* expression with the abundance of six types of TIICs in the TIMER database. *ENTPD1*, extracellular nucleotide triphosphate diphosphate-1; ESCA, esophageal carcinoma; TIICs, tumor-infiltrating immune cells; rho, correlation coefficient; cor, correlation coefficient. [a] Spearman's rank correlation was performed.

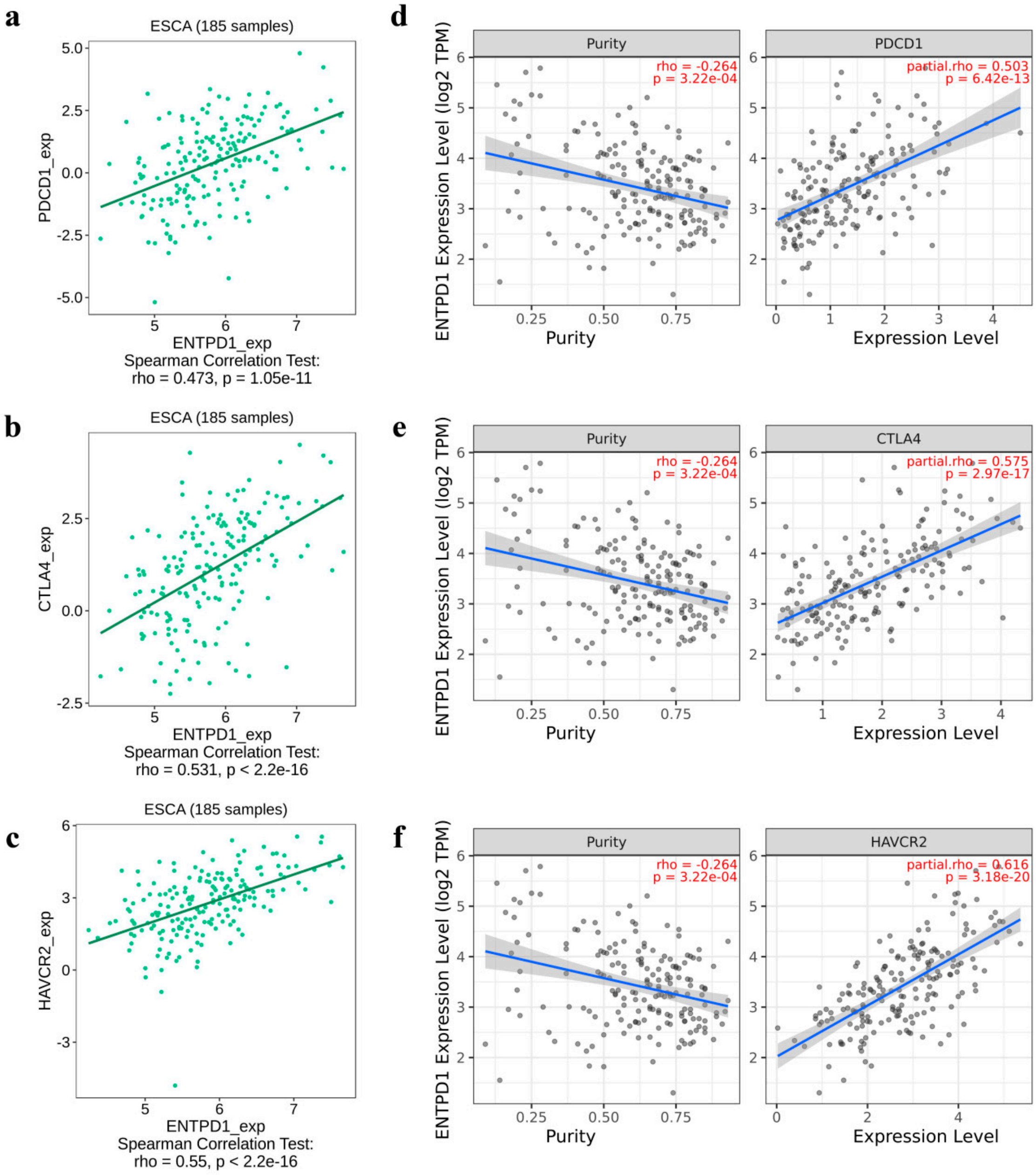

Figure S2. Correlation of *ENTPD1* expression with the expression of inhibitory immune checkpoint molecules in ESCA [a]. (**a-c**) Correlation of *ENTPD1* expression with the expression of *PDCD1*, *CTLA4* and *HAVCR2* in the TISIDB database; (**d-f**) Correlation of *ENTPD1* expression with the expression of *PDCD1*, *CTLA4* and *HAVCR2* in the TIMER2.0 database**.** *CTLA4*, cytotoxic T-lymphocyte-associated protein 4; *ENTPD1*, extracellular nucleotide triphosphate diphosphatehydrolase-1; ESCA, esophageal carcinoma; rho, correlation coefficient; *HAVCR2*, hepatitis A virus cellular receptor 2; *PDCD1*, programmed cell death protein 1. [a] Spearman's rank correlation was performed.
